# Small Bowel Prolapse a Rare Complication Following Unsafe Abortion

**DOI:** 10.7759/cureus.11260

**Published:** 2020-10-30

**Authors:** Subha Ranjan Samantray, Ipsita Mohapatra

**Affiliations:** 1 Obstetrics and Gynecology, Prathima Institute of Medical Sciences, Karimnagar, IND

**Keywords:** abortion, unsafe abortion, bowel prolapse, bowel injury, dilatation and curettage

## Abstract

Small bowel prolapse through uterine perforation is a rare but severe complication of unsafe abortion. Early recognition of the bowel prolapse, aggressive resuscitation and prompt surgical intervention can reduce the morbidity and mortality related to these kinds of injuries. We present here a case of small intestine prolapse through uterine perforation following dilatation and curettage requiring intestinal resection. Efforts have to be made to reduce the number of unsafe abortions.

## Introduction

A procedure for termination of an unintended pregnancy carried out by either person lacking the necessary skills or in an environment which does not conform to minimal medical standards or both is defined as unsafe abortion [1]. Abortion may be spontaneous or induced. The indications of induced abortion are various like unwanted pregnancies, economic, social or family problems, health problems of the mother or any severe congenital defect of the foetus [2]. Medical or surgical methods can do induction of abortion. Amongst the surgical methods, cervical dilatation and curettage (D&C) is the most commonly performed procedure. D&C is a safe method of termination in the early trimester of pregnancy. However, sometimes uterine perforation may occur. Bowel injury through this perforation is rare, but a severe complication. Bowel prolapse through this perforation is even rarer and always needs laparotomy to save the life of the patient. We present here a case of small intestine prolapse through uterine perforation following D&C done by a non-medical practitioner.

## Case presentation

A 23-year-old woman, presented to our hospital with complaints of severe abdominal pain and something coming out through the vagina. On history taking, it was revealed that she had three months of pregnancy and had gone to a local non-medical practitioner for termination of pregnancy. She had one previous normal delivery one year back. She underwent termination of pregnancy in her village. During the procedure, the non-medical practitioner observed something coming out through her vagina and referred the patient to our hospital on the same day.

**Figure 1:**
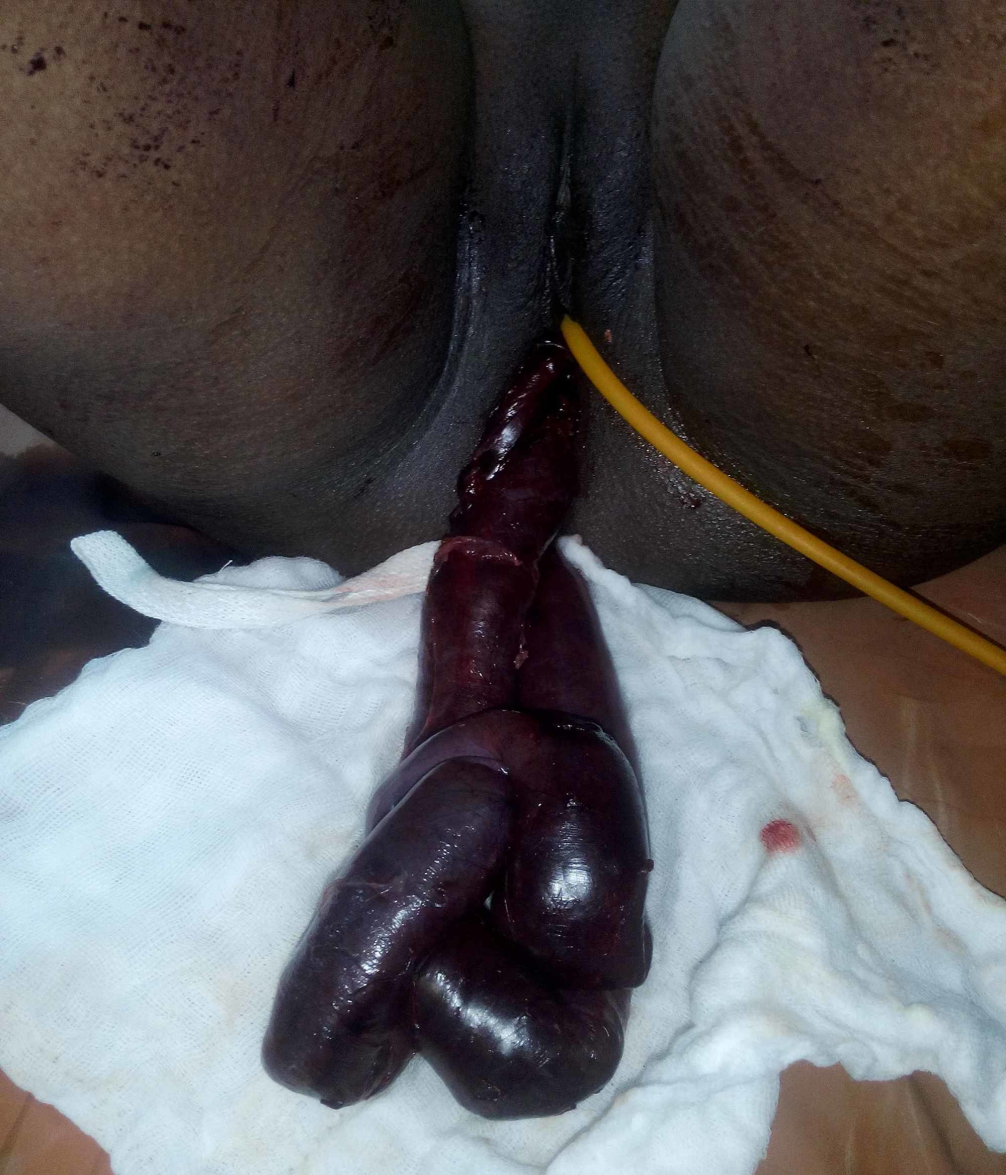
Prolapsed bowel segment through the vagina

On examination, her vitals were impaired. Blood pressure was 90/70 mm of Hg, and pulse was 106/ min. The temperature was normal, 98.4ᴼF. On general examination, pallor was present. Her abdominal examination was difficult to appreciate due to the presence of tenderness, muscle guarding and rigidity. Bowel sounds were sluggish. Inspection of vulva revealed prolapsed bowel loops. The length of the visible prolapsed bowel segment was about 40 cm. On per speculum examination, we could see the loop of intestine coming out through the cervix. It was bluish coloured, and some of its portions appeared gangrenous (figure 1).

**Figure 2:**
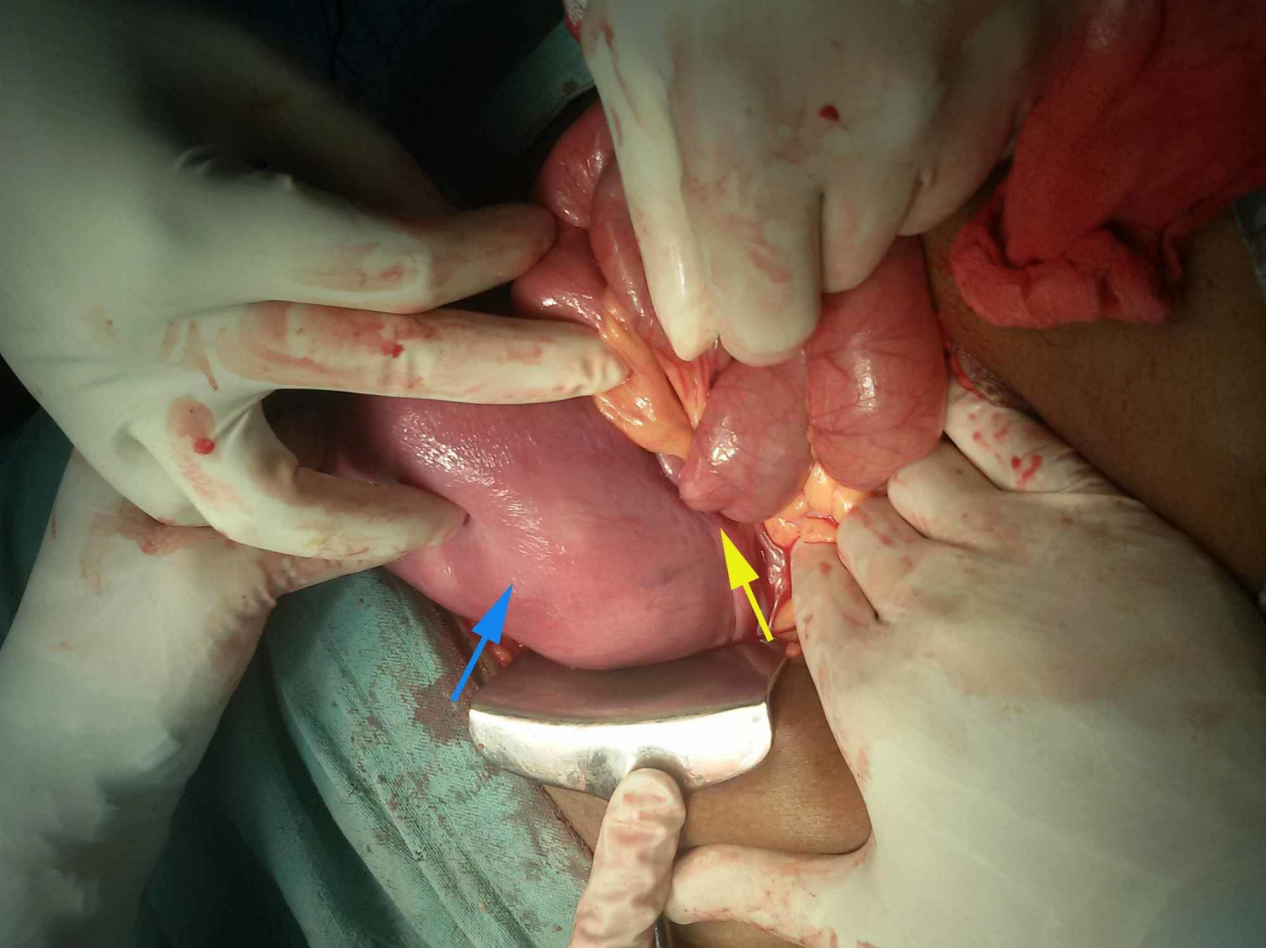
Bowel segment entering uterine rent Uterus( blue arrow), Rent on the left lateral wall of the uterus( yellow arrow)

**Figure 3:**
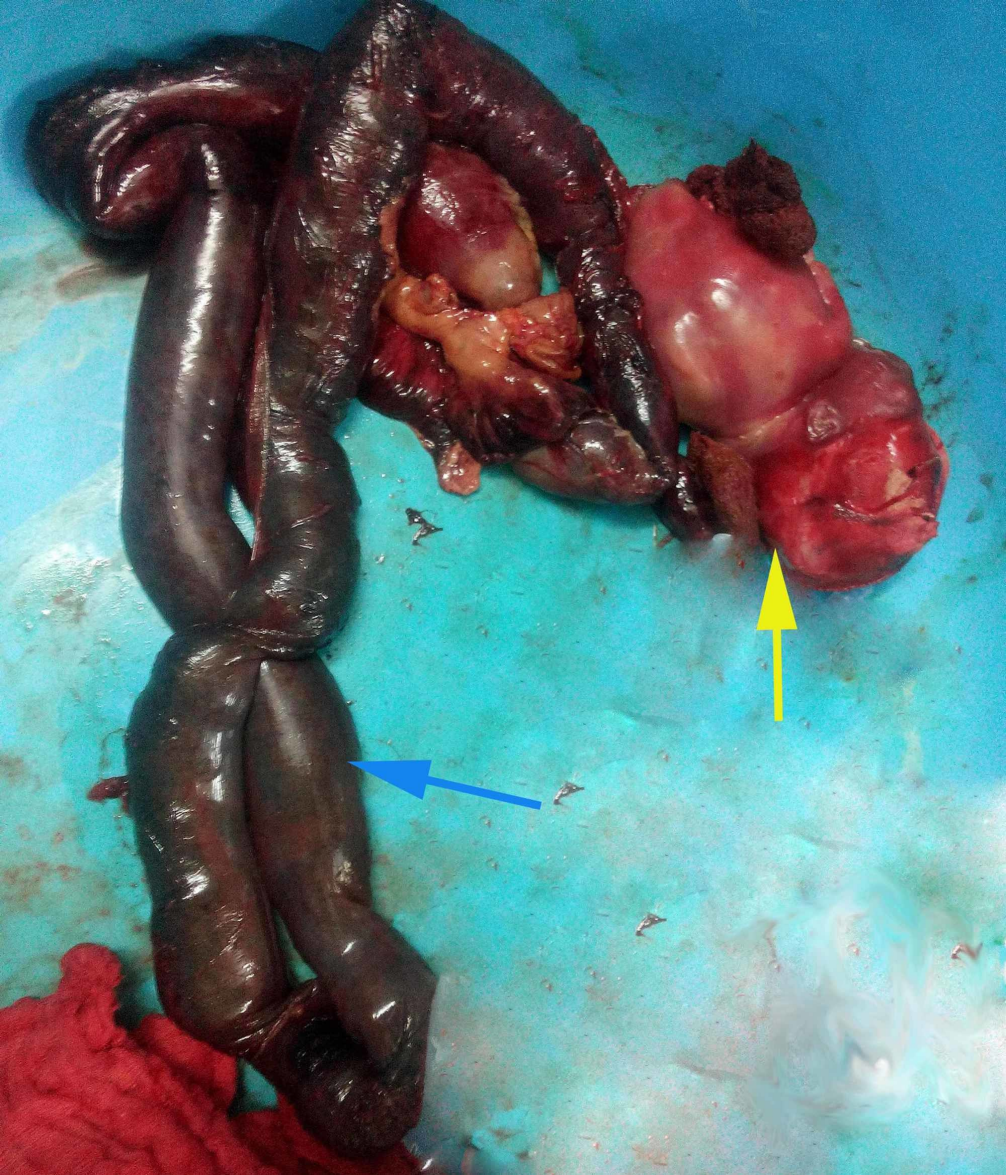
Resected bowel segment with the foetus Resected bowel segment( blue arrow), Part of the foetus (yellow arrow)

The patient was resuscitated. After doing proper workup and routine investigations, an exploratory laparotomy was planned. Per operative, findings revealed a rent of about 3×3 cm in the left anterolateral wall in the body of the uterus, through which the ileum was seen entering into the cavity (figure 2). The uterine rent was enlarged. Through this rent, the prolapsed bowel segment was gently pulled into the abdominal cavity. A portion of this ileal segment was found to be detached from its mesentery, appeared non-viable and gangrenous (approximately 50 cm in length). This segment was resected and end to end ileo-ileal anastomosis was done. Digital evacuation of uterine contents was done through the rent. The products of conception, including the foetus, were removed and then the rent was repaired in two layers using vicryl (polyglactin 910) no-1 suture (figure 3). Thorough lavage of the peritoneal cavity was done with warm normal saline and metronidazole. Intraperitoneal drain was kept. Postoperatively she was put on intravenous piperacillin-tazobactam 4.5gm every 8hr, metronidazole 500mg every 8 hr and amikacin 500mg every 12hr for five days. Gastric aspiration was continuously done for 48 hours. A drain was removed on the fourth postoperative day. Sutures were removed on the ninth postoperative day, and she was discharged on 10th postoperative day in stable condition.

 

## Discussion

Termination of pregnancy by medical methods carried out in places outside health facilities, without a prescription accounts for about 70% of all abortions in India, amongst which 5% underwent highly unsafe methods with a high risk of complications [3]. In developing countries like India, though abortion is legalised, it is still inaccessible to the majority of the women living in remote areas. Consequently, they may approach local dais or untrained persons lacking the required knowledge and skill for termination of unwanted pregnancies. Unsafe abortions are one of the most ignored reproductive health hazard associated with potential complications like haemorrhage, infections, uterine perforation, visceral injuries, chronic pelvic pain and secondary infertility.

The risk factors associated with increased chances of uterine perforation are previous D&C, prior caesarean section, advanced maternal age, greater parity, and lack proper training of the persons carrying out the procedure [4]. Globally the reported incidence of uterine perforation varies from 0.4 to 15 per 1000 abortion [5]. Bowel perforation is a rare but severe complication of D&C [6]. Chawla S et al. reported the incidence of intestinal injury to be ranging between 5-18% [7]. The ileum and sigmoid colon are more vulnerable to injury due to their close relationship to the posterior surface of the uterus [8]. Bowel may be injured during the procedure by a curette, ovum forceps, plastic cannula or even by a uterine sound. Injury can result in perforation, contusion, hematoma or complete transection of the bowel requiring emergency laparotomy. Bowel prolapse through uterine perforation is even rarer. Augustin G et al. published a systematic review of 73 articles in which 12 out of 30 cases described bowel prolapse as a complication of surgical abortion [4].

The patients with uterine perforation following surgical abortion may present with vaginal discharge, pain, abdominal cramps or fever. Bowel injury secondary to uterine perforation can lead to adhesions, partial or complete obstruction leading to abdominal pain with/without distension, vomiting, diarrhoea or absence of flatus or stool [4]. If left untreated, it may end in peritonitis, septic shock and even death [9]. Rarely bowel can prolapse through the uterine rent into the vagina and can be seen outside the vulva. Bowel prolapse is a medical emergency due to which the patient may deteriorate rapidly.

Uterine perforation following surgical termination can have life-threatening complications [10]. However, diagnosis and management at the correct time may be life-saving. Uterine perforation can be suspected when there is a sudden loss of resistance while performing the procedure. Ultrasound can detect a defect in the uterine wall. When the patient presents with non-specific symptoms like abdominal distension, abdominal X-ray can show air-fluid levels of small bowel to point towards obstruction. Ultrasound examination showing free fluid in pelvis and loops of intestine within the endometrial cavity can clinch the diagnosis of bowel involvement [11]. Computed tomography scan can also help in diagnosing bowel loops within the endometrial cavity when ultrasound report is ambiguous.

The most critical factor in the prevention of this condition is the identification of patients who are at high risk of perforation. D&C should always be done under ultrasonographic guidance whenever available [11,12]. Early diagnosis, prompt resuscitation and treatment can prevent peritonitis, septic shock or any other severe complications to the patient. Bowel injury following D&C almost always needs operative intervention. The prolapsed bowel segment needs to be gently pulled back into the abdominal cavity, and the viability of this segment has to be established. If the patient presents early (within 24 hours), primary repair of the damaged intestinal segment can be done as there are chances of minimal contamination [2]. However, with late presentation (after 24hr), ileostomy and reanastomosis of two segments may be needed. Our patient presented early on the day of D&C, and hence primary repair by ileo-ileal anastomosis was possible. Patients are followed up with ultrasound and serum β-subunit of human chorionic gonadotropin (beta hCG) to rule out retained products of conception.

## Conclusions

Complications like intestinal injury due to unsafe abortion are rare but dreaded. Early recognition of the injury, aggressive resuscitation and prompt surgical intervention can reduce the morbidity and mortality related to these kinds of injuries. Efforts have to be made to reduce the number of unsafe abortions. Women should be counselled for contraception, family planning, and they should be made aware of the various contraceptive choices. There should be decentralisation of the health care facilities, so that rural population do not opt for illegal methods of termination of pregnancy. The local health providers should be given basic training so that they can diagnose and refer the patient to a tertiary care centre if any untoward hitches occur during the procedure.
